# Supplementary material for: Iron Supplementation Increases Tumor Burden and Alters Protein Expression in a Mouse Model of Human Intestinal Cancer
Source: Nutrients. 2024 Apr 27;16(9):1316. doi: 10.3390/nu16091316 (PMC11085868; doi:10.3390/nu16091316)
Supplement: Supplementary file 1 [file nutrients-16-01316-s001.zip › nutrients-2974622-supplementary.pdf]

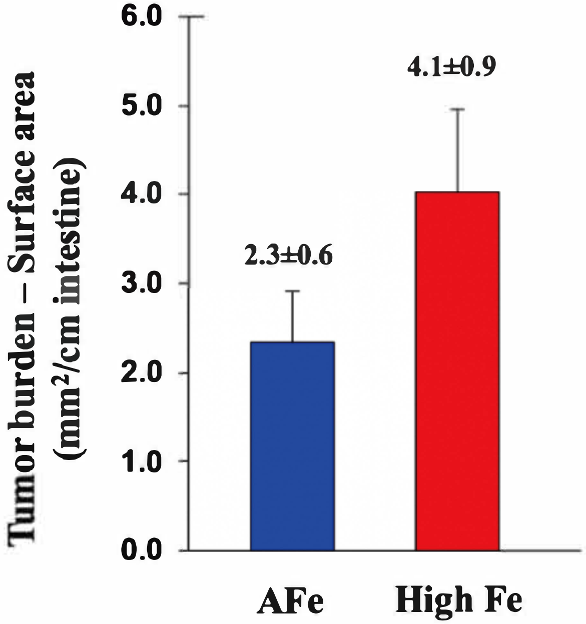

Figure S1. Tumor burden (mm$^{2/}$cm) in intestines of mice fed adequate and excess iron, at wk 10.

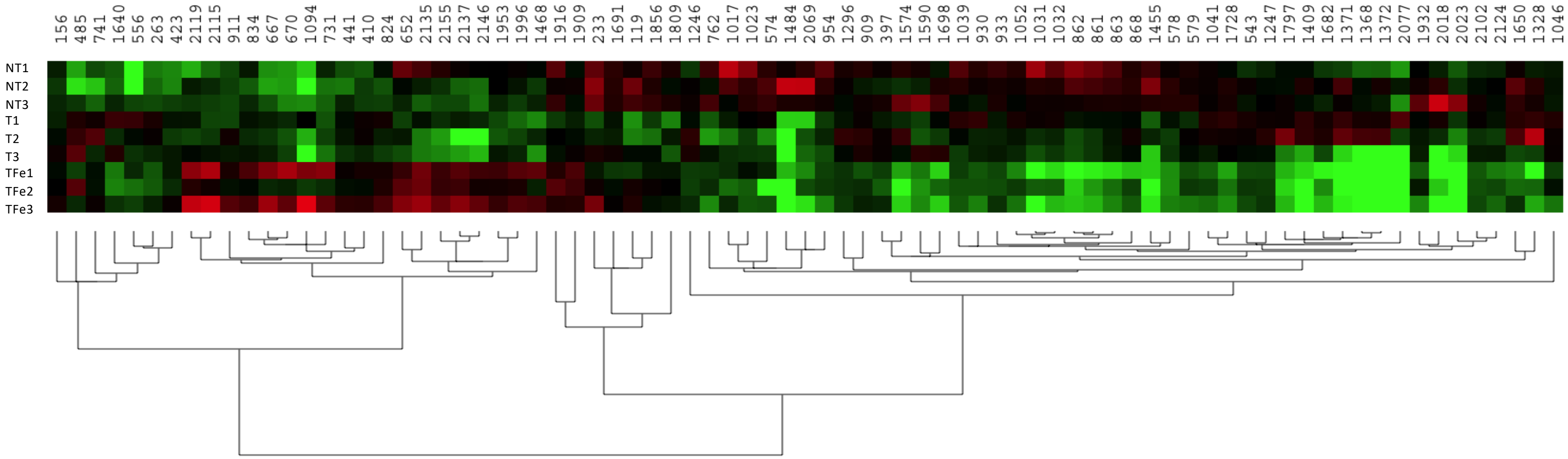

Figure S2. Hierarchical clustering of differentially expressed protein spots in tumor (T) and adjacent non-tumor (NT) intestinal tissue. Color differences illustrate log-transformed volume of protein spots; Samples, in triplicate, are from the different experimental groups are indicated on the column labels, with protein spots numbers displayed on the y-axis. Red color indicates high level expression, green color indicates low expression, and black indicates median expression. (Key: NT and T = normal and tumor tissues from the adequate iron group; TFe = tumor tissue from the excess iron group.)

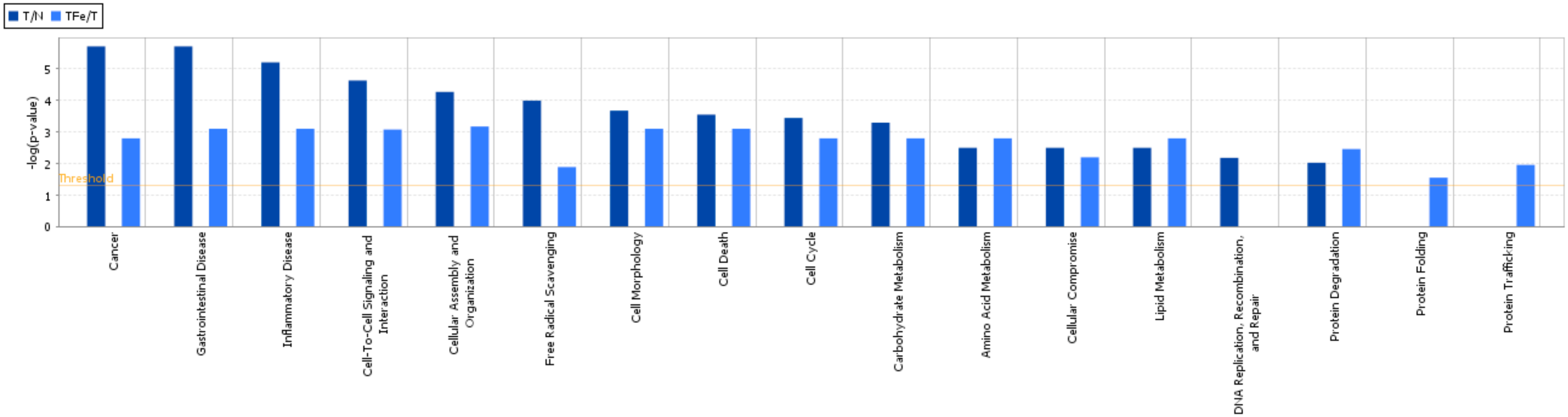

Figure S3. Using Ingenuity Pathways Analysis, we found 4 highly significant functional networks in tumor as compared to normal tissue and 2 in tumors from mice fed adequate as compared to high iron. Notably, the top network in both cases was associated with functions implicated in cancer and cell death. (Key: "T/NT" = 45 mg/kg dietary iron tumor vs. non-tumor tissue; "TFe/T" = 450 mg/kg dietary iron tumor vs. 45 mg/kg dietary iron tumor tissue).
